# Failure modes and mechanisms of layered h-BN under local energy injection

**DOI:** 10.1038/s41598-022-16199-y

**Published:** 2022-07-13

**Authors:** Ping Liu, Qing-Xiang Pei, Yong-Wei Zhang

**Affiliations:** Institute of High Performance Computing, A*STAR, Singapore, 138432 Singapore

**Keywords:** Materials science, Physics

## Abstract

Layered h-BN may serve as an important dielectric and thermal management material in the next-generation nanoelectronics, in which its interactions with electron beam play an important role in device performance and reliability. Previous studies report variations in the failure strength and mode. In this study, using molecular dynamics simulations, we study the effect of local heat injection due to the electron beam and h-BN interaction on the failure start time and failure mode. It is found that at the same heat injection rate, the failure start time decreases with the increase in the layer number. With the introduction of point defects in the heating zone, the failure always starts from the defect site, and the start time can be significantly shortened. For monolayer h-BN, failure always starts within the layer, and once failure starts, its propagation is through melting or vaporization of the h-BN atoms, and no swelling occurs. For multiple layers, once failure starts within the h-BN film, swelling occurs first. With continued heating, the large pressure induced by melting and vaporization can cause the burst of the layers above, leading to the formation of a pit. In the presence of multiple defects within the heating zone, these defects can interact, causing a further reduction in the failure start time. We also reveal the relation of beam power with layer-by-layer failure mode and swelling/pit formation mode. The present work not only reproduces many interesting experimental observations, but also reveal several interesting mechanisms responsible for the failure processes and modes. It is expected that the findings revealed here may provide useful references for the design and engineering of h-BN for device applications.

## Introduction

The interactions of electron beam or laser beam with materials play an important role in modern technologies, ranging from modulating device functionality, property characterization, processing and manufacturing, et al.^1,2^. For example, due to the heat generated by electron beam and laser beam, melting can occur in powder, which then can be used to build a structure or device layer by layer, that is, additive manufacturing. Another example is the use of electron beam or laser beam for surface patterning and treatment. Perhaps the most well-known application of electron beam is the use in scanning electron microscopy or transmission electron microscopy for microstructural imaging and characterization of materials.

Through shining an electron beam or laser beam on a material surface, electrons or photons will interact with the material, which may cause changes in the mass, energy, composition or structure of the material^1,2^. Depending on the type of materials, the responses can be quite different. Some materials can strongly absorb the beam energy while others may be transparent. Also, depending on the intensity of the electron beam or laser beam, the responses of a material can also be quite different. When the beam intensity is low, such beam often does not cause any atomic structure changes. With the increase of energy intensity, local structure changes may occur, which can cause defect formations. When the intensity is further increased, melting or even vaporization may occur, causing the breakup of the material structure and functionality^1,2^. A well-known example is the dielectric breakdown (DB) under an electric field^3^.

2D materials are an emerging class of nanostructured low-dimensional materials with great promise in serving as building blocks for the next generation nanoelectronic devices and engineering applications^4–6^. Among the 2D materials family, 2D h-BN, which is an insulator with mechanical robustness, thermal stability and chemical inertness, is one of the most promising materials for many technological applications ranging from optics to electronics^7^. Due to its attractive properties, it is promising to use it as high-performance dielectric material in high-power electronic devices. However, such applications require its interactions with high-energy electron beam, which can strongly influence its structural integrity and functionality^8–11^.

It is noted that several studies have been carried out to understand the failure processes and mechanisms of h-BN under an electron beam^8–14^. For example, Lee et al. investigated the electron tunnelling through mono-, bi-, and tri-layer h-BN, and found that Flowler–Nordheim tunnelling occurred at high bias. They also observed an increase in DB voltage with the h-BN film thickness^8^. Hattori et al. investigated the DB characteristics of h-BN using conductive atomic force microscopy (c-AFM). They observed that the breakdown of the h-BN multiplayer begins in the top layer and then proceeds layer-by-layer caused by physical fracture^9^. The layer-by-layer failure pattern was also observed by Ji et al.^10^. Ranjan et al. showed that defect clusters were present in their h-BN films which were likely due to thickness variations and microstructural inhomogeneity, and boron vacancies were found to be the critical defect type that assisted the overall charge transport and DB in the h-BN films^11^. Jiang et al.^12^ performed c-AFM analysis on h-BN films with varying thicknesses. They found that when DB was triggered, multilayer h-BN stacks showed severe surface extrusion (hillock formation), while the monolayer h-BN did not show such surface extrusion. Ranjan et al. showed that the defect generation and dielectric degradation in h-BN films could occur through progressive breakdown (PBD) and hard breakdown (HBD). In the PBD stage, defects are generated progressively in the films. While in the HBD, h-BN materials are removed physically from the films, leading to the formation of pits at the breakdown locations^13,14^.

These previous studies clearly show the influences of film thickness (or layer number) and defects on the structural failure and dielectric breakdown. It is intriguing to notice the conflicting results from these studies. For example, layer-by-layer breakdown was observed, causing the hole or pit formation. While surface extrusion rather than hole or pit formation was observed in h-BN films. Also the role of defects in the breakdown is evident. However, whether it is the pre-existing defects formed during the growth or transfer^15,16^ or defects nucleated under the electron beam is not clear. Last but not least, failure patterns in monolayer and multilayer are also different. It would be interesting and important to understand underlying reasons for these differences.

In this work, we perform molecular dynamics simulations to examine the failure processes and mechanisms of h-BN films with varying thicknesses and defect concentrations and distributions under local energy injection. The local energy injection is the consequence of energy absorption of electron beam by the h-BN film. Our simulations show that for defect-free h-BN films, the failure start time decreases with the increase of film thickness under the same energy injection rate. When B vacancy is introduced into h-BN layers, however, this trend can be significantly altered. In the presence of existing defects, the films always start the failure at the defect sites. When the defect site is away from the surface layer, surface extrusion due to melting and vaporization around the defect may occur. Under further electron beam exposure, the large pressure from the vaporization can cause the h-BN top layers to fracture, leading to the formation of pits. However, when the defect density is high and defects distributed in different layers could interact with each other, causing a further reduction in failure start time. These simulations explain the different failure patterns observed in previous experiments. We further analyse factors, such as the boundary conditions and energy injection rate, in influencing the h-BN failure processes and suggest possible strategies to alleviate the breakdown of h-BN films.

## Simulation models and methods

**Figure 1:**
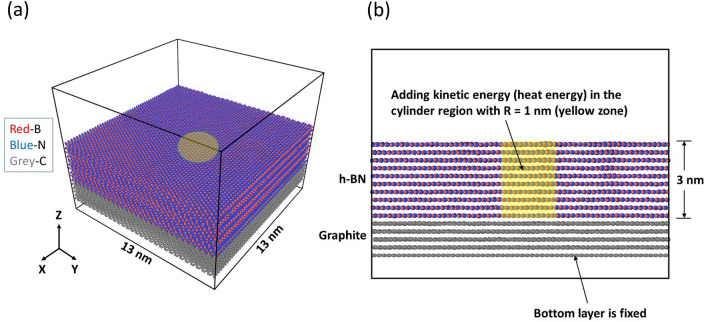
Schematic illustration of lattice structures and simulation model. (**a**) The 3D simulation model. (**b**) The cross-section of the simulation model. The yellow zone is the energy exchange due to the collisions between electrons and h-BN atoms.

The simulation model is schematically shown in Fig. 1a (3D view) and Fig. 1b (*yz* cross-section view), in which the h-BN film is overlying on a multilayer graphene substrate. In the present consideration, the lateral dimensions of the model are 13 nm by 13 nm, and the number of h-BN layers varies from 1 to 10 layers. For multilayer h-BN, the thermodynamically stable AB stacking scheme is taken. Here, the multilayer graphene is a five-layer film also with the AB stacking scheme. In the simulations, the bottom graphene layer is fixed. Here, it is assumed the electron beam shines vertically on the h-BN film.

During the interaction between electrons and h-BN layers, the energy carried by electrons is transferred to h-BN lattice vibrations or phonons. Since graphene is a conductor while h-BN is an insulator, it is expected that the electrical conductivity of graphene is much higher than that of h-BN. In addition, both the in-plane and out-of-plane thermal conductivities of graphene are much higher than those of h-BN (Table 1), it is expected that heat generation (Joule heating) in graphene will be negligible in comparison with that in h-BN. As a result, the intensive heat can be generated in the h-BN. Since the heat distribution is dependent on many factors, such as beam intensity, beam flux distribution, beam and h-BN interaction, here we do not explicitly consider the interaction processes of electrons and h-BN film, but instead, consider the heat generation arising from the beam and h-BN interaction. Therefore, we focus on the effect of heat generation in the h-BN film due to its interaction with electrons. For simplicity, we assume the heat injection zone takes a cylindrical shape with radius R = 1 nm as shown in Fig. 1a,b.

**Table 1:** Thermal conductivity (300 K) of h-BN and graphene.

Materials	Materials type	Sample type	Orientation	Thermal conductivity (W/m/K)	References
h-BN	Insulator	Monolayer	In-plane	600	^17^
		5 layers	In-plane	250	^18^
		11 layers	In-plane	360	^18^
		Pyrolytic h-BN	Out-of-plane	2	^19^
Graphene	Conductor	Monolayer	In-plane	4840–5300	^20^
		Monolayer	In-pane	5000	^21^
		Monolayer	In-plane	3080–5150	^22^
		Graphite	Out-of-plane	6	^23^

A typical heat injection rate of 0.254 eV/atom/ps is taken unless stated otherwise. Here it is assumed that both the heat injection rate for Boron and Nitrogen atoms is the same. The injected heat can diffuse out of the heating zone along the in-plane h-BN due to the good in-plane thermal conductivity of h-BN, and across the interface between h-BN and graphene into graphene since the interfacial thermal conductance of 135 MWK^−1^ m^−2^ enables efficient heat transport across the interface^24^.

To model the h-BN/Graphene heterostructure, a free boundary condition is applied to the h-BN top layer (positive *z* direction) and a periodic boundary condition along the *x* and *y* directions. Molecular dynamics (MD) simulations are employed to study the energy injection and thermal transport across the h-BN/Graphene heterostructures, and failure processes using the LAMMPS package^25^.

In all MD simulations performed here, the Tersoff potential^26,27^ is used to describe the covalent interactions between C, B and N atoms in each layer of h-BN and graphene with optimized parameters for thermal and mechanical properties^28–30^. The interlayer van der Waals interactions are described by the Lennard–Jones (LJ) potential. The LJ parameter values are obtained from the universal force field (UFF) by Rappe et al.^31^. The simulations are carried out with a time step of 0.5 fs throughout. The velocity Verlet algorithm is employed to integrate Newton’s equations of atomic motion. First, the system is equilibrated at a constant temperature of T = 300 K for 200 ps using Nosé–Hoover temperature thermostat^32^ (*NVT* ensemble). After the constant temperature relaxation, the heat energy is continuously injected into the heating zone. During this stage, the atomic energy and temperature in the system are monitored.

In real h-BN samples, various defects, such as vacancies, dislocations, and grain boundaries, can be present. These defects may influence the failure processes and mechanisms. A previous study identified B vacancies to be the critical defect type for dielectric breakdown failure^13^. Also another previous study^15^ showed that B vacancy density (about 1.1 × 10^5^ per μm^2^) can be two orders of magnitude higher than that of other defects. Hence, B vacancies are the absolutely dominating defect type in h-BN.

In the present work, since the radius of the heat injection zone is 1 nm, the energy injection lateral area is 3.14 nm^2^. Since the vacancy density is about 1.1 × 10^5^ per μm^2^, the number of vacancies within one layer in the energy injection zone is about 0.3454. Hence, we take 1 in the present work. To study the interaction between defects, we placed defects in different layers and yet close to each other to study the effects of their interactions.

To consider the presence of intrinsic defects in the h-BN^15,16^, we introduce both B vacancies and N vacancies into the heating zone of the h-BN film. The effects of different locations and distributions of defects are also examined.

## Results and discussion

Below, we would like to systematically investigate and understand the effects of h-BN layer number and defect type, location and distribution on the failure start time and location of the h-BN/Graphene heterostructure.

### Defect-free h-BN

**Figure 2:**
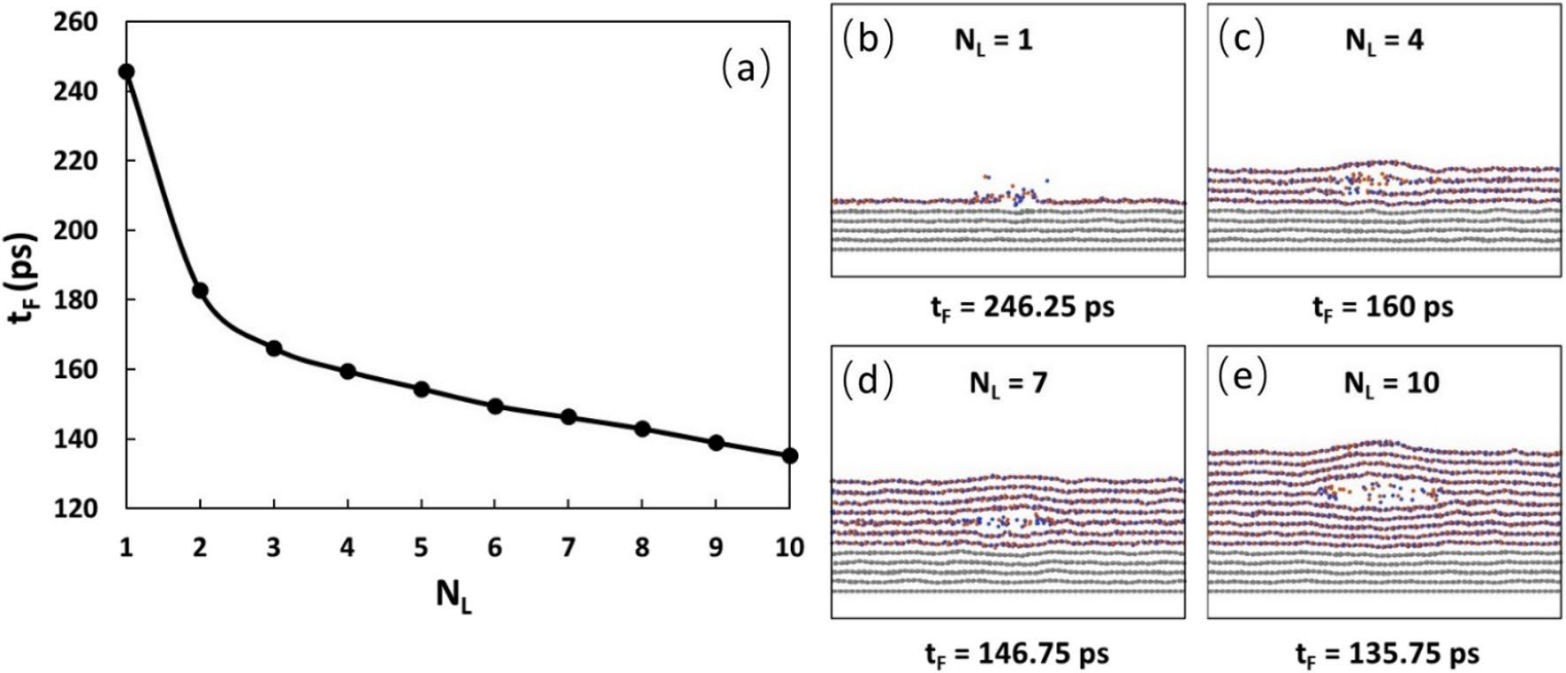
(**a**) Variation of failure start time with the number of h-BN layers. (**b**–**e**) Failure location for N_L_ = 1, 4, 7, 10, respectively. For the failure start time, the general trend is that the thinner the film, the longer the failure start time. For the failure location, the general trend is that for thin h-BN films (N_L_ = 1, 2, 3), the failure starts at the top layer. For thick thickness (N_L_ > 4), the failure starts in one of the middle layers.

To understand the failure processes and mechanisms of defect-free h-BN under the heat injection, we first examine the effect of the number of h-BN layers, N_L_. Variations of failure start time t_F_ and location with the number of h-BN layers are shown in Fig. 2. It is observed that the thinner the h-BN film, the longer the failure start time (Fig. 2a), indicating that a thin h-BN will have a higher heat failure resistance. For example, for N_L_ = 1, the failure start time is 246.25 ps while for N_L_ = 2, the failure start time drastically reduces to 182.75 ps. When the N_L_ is larger than 3, the failure start time decreases approximately linearly with the number of h-BN layer. At N_L_ = 10, the failure start time reduces to 135.75 ps. Although the failure start position always falls in the heating zone, the failure start location appears to depend on the h-BN film thickness. The general trend is that for the thin h-BN films (N_L_ = 1, 2, 3), the failure starts at the top layer (Fig. 2b), while for the thick films (N_L_ > 3), the failure starts in one of the middle h-BN layers. For example, for N_L_ = 4, the failure starts at the second layer (Fig. 2c), while for N_L_ = 7, the failure starts at the fifth layer (Fig. 2d) and for N_L_ = 10, the failure also starts at the fifth layer (Fig. 2e). Clearly, failure location is dependent on the film thickness.

The underlying mechanism for this thickness-dependent failure can be explained by two factors, that is, the geometry constraint effect and interfacial thermal conduction effect. For the thin h-BN films, due to the efficient interfacial thermal conduction between h-BN and graphene, the heat generated in the h-BN heating zone can be efficiently transported into the graphene substrate, reducing the failure probability for the layers near the graphene substrate. Also, since the top layer is lack of geometrical constraint from its top, the failure is expected to occur in the top layer. For the thick films, the large heat generated in the center of the heat zone is unable to efficiently diffuse out, leading to a fast heat accumulation there, causing the failure to start in one of the middle layers.

### Effects of B and N single vacancy

**Figure 3:**
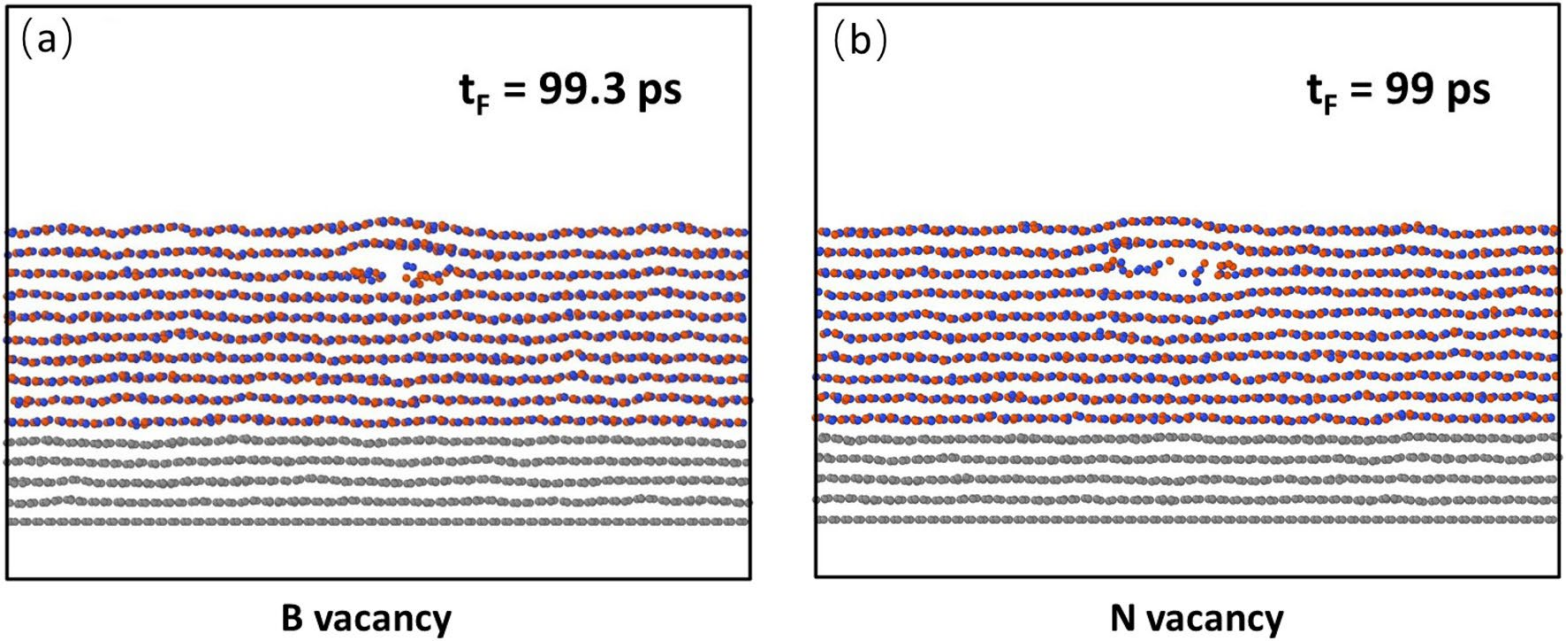
Effect of vacancy on the failure start time and location. (**a**) B vacancy and (**b**) N vacancy. In both cases, the vacancy is located in the middle of the 3rd layer of the heating zone, and the failure starts from the defect. The failure start time for B vacancy is 99.3 ps and that for N vacancy is 99 ps, indicating that there are almost no differences in the failure time and failure location for both types of defects. Both failure start times are much shorter than that of defect-free case, which is 135.75 ps.

It is known that h-BN possesses high vacancy formation energies for both B and N (7.65 eV and 8.47 eV, respectively^33^). The high migration energies for B and N are also high (2.6 eV and 5.8 eV, respectively^34^). Since the formation energy and migration energy of N vacancy are higher than those of B vacancy, it is expected the concentration of N vacancy should be higher than that of B vacancy. To examine the effects of B and N single vacancy in the heating zone on the failure behaviour of the heterostructure, we take the sample with 10 h-BN layers and insert a B and N vacancy, respectively, in the middle of the 3rd layer of the h-BN film. The failure processes are shown in Fig. 3a for B vacancy and Fig. 3b for N vacancy. It is seen that in both cases, the failure starts from the defect site. The failure start time for B vacancy is 99.3 ps and that for N vacancy is 99 ps, indicating that there is almost no difference in the failure time for both types of defects.

The mechanism for this indifference can be explained by the fact that at a B vacancy, each of the neighboring three N atoms bonds with two B atoms. The rapture of the two B–N bonds is required to make the failure propagate. At a N vacancy, each of the neighboring three B atoms bonds with two N atoms. Hence, the rapture of two N–B bonds is required to make the failure propagate. Since both require the rapture of two N–B bonds for failure to propagate, this explains why there is almost no difference in the failure start time for both cases. The failure start times for both cases are, however, much shorter than that of defect-free case, which is 135.75 ps. Since the difference in the failure start time and location for both types of defects are almost the same, in the following, we only consider the B vacancy.

### Effect of heat injection rate

**Figure 4:**
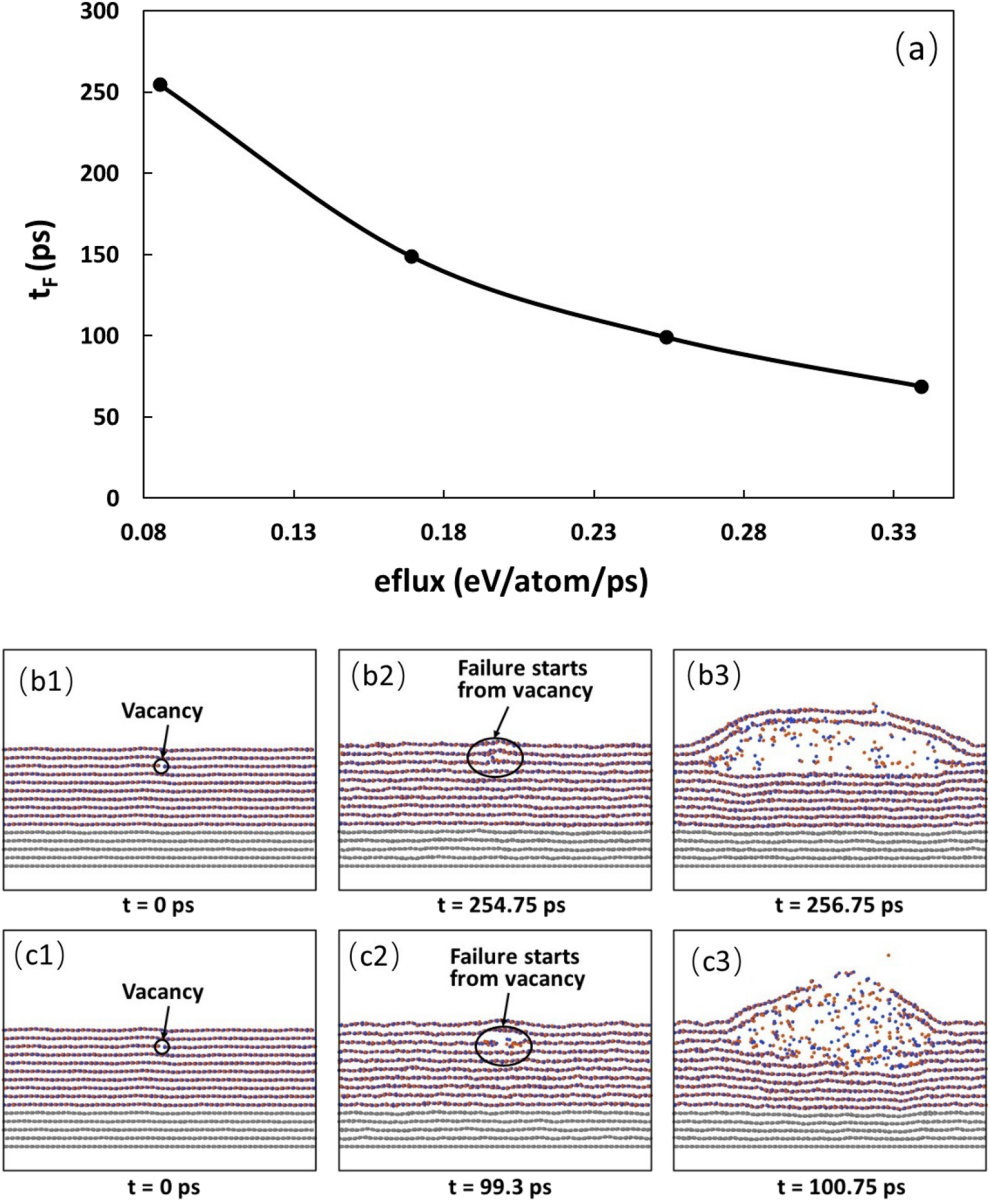
(**a**) Variation of failure start time with the heating rate. In the simulations, the sample with 10 h-BN layers is used with B vacancy in the middle of the 3rd layer. (**b1**–**b3**) Failure of the sample with the heating rate of 0.085 eV/atom/ps. (**c1**–**c3**) Failure of the sample with the heating rate of 0.254 eV/atom/ps.

In the above, we have taken the heat injection rate to be 0.254 eV/atom/ps. However, different heat injection rates may lead to different heat accumulation and transport processes, and thus different failure start times and failure modes. It is expected that a higher heating rate will lead to a short failure start time and vice versa. Here, we compare the failure processes under four different heating rates, that is, 0.085, 0.169, 0.254, and 0.339 eV/atom/ps. The variation of failure start time with the heating rate is shown in Fig. 4a and the failure processes for the heating rate of 0.085 and 0.254 eV/atom/ps are shown in Fig. 4b1–b3 and c1–c3, respectively.

It is seen that with the increase of the heating rate, the failure start time decreases, which is expected as a higher heating rate will cause a faster heat accumulation in the heating zone while the heat diffusion out of the heating zone cannot catch up with the heat generation, thus causing the earlier failure start time. It is also noted that this decrease of failure start time follows a sublinear relation, indicating that with the increase in the heating rate, the heat diffusion also increases. However, the heat diffusion out of the heat zone cannot match up with the heat generation in the heat zone, causing the sublinear relationship.

### Defect location

**Figure 5:**
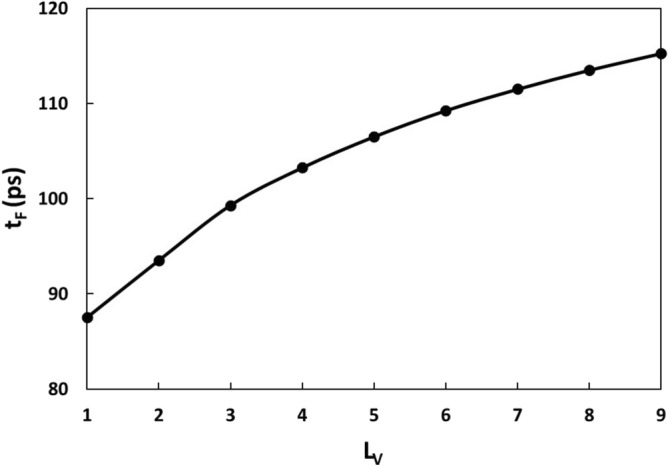
Variation of failure start time with the B vacancy position. L_v_ is the number of the h-BN layer counting from the top layer with a B vacancy.

**Figure 6:**
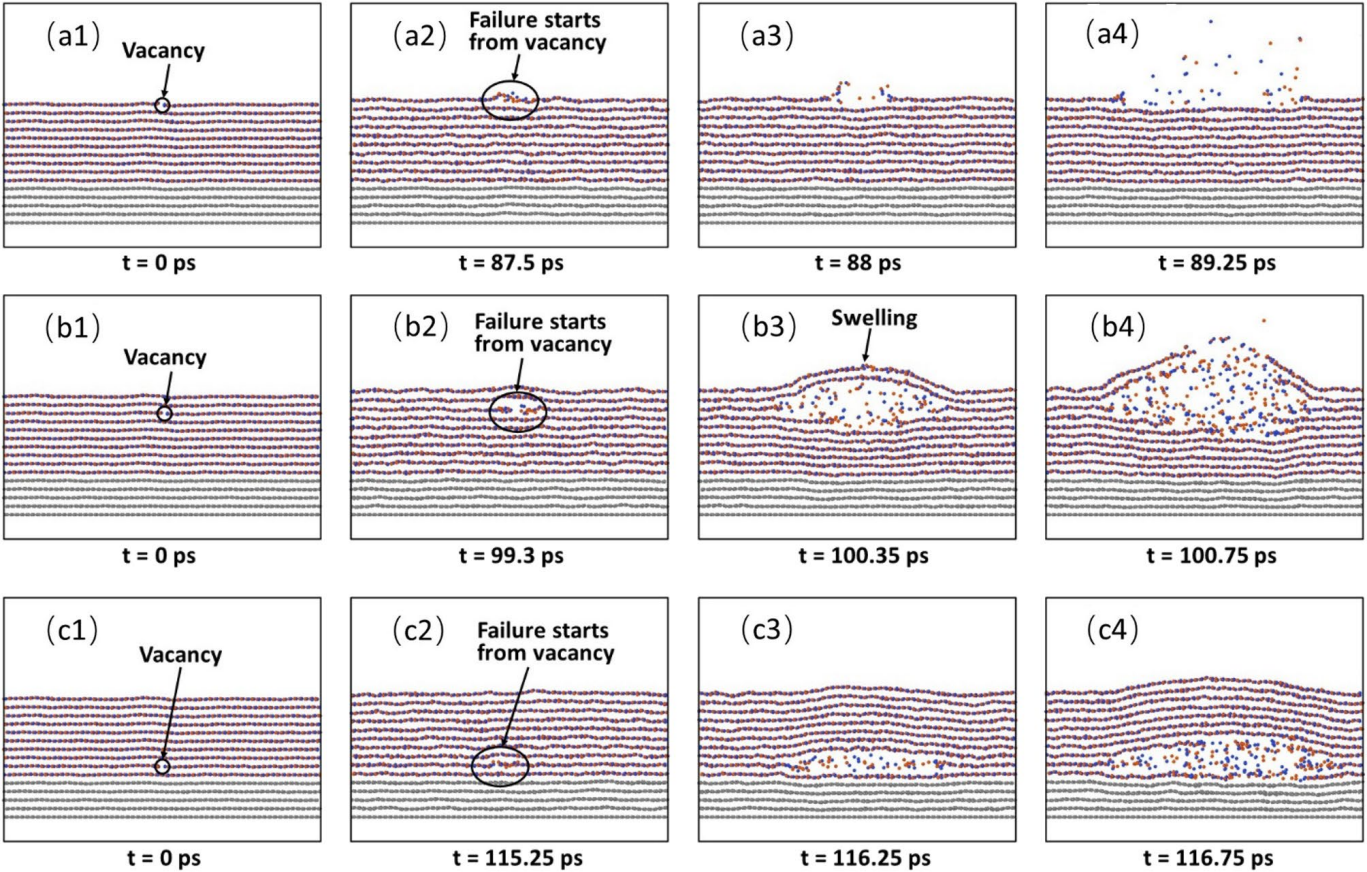
Snapshots of failure processes for a single B vacancy at a different h-BN layer. (**a1**–**a4**) B vacancy at the top layer. (**b1**–**b4**) B vacancy at the 3rd h-BN layer. (**c1**–**c4**) B vacancy at the 9th h-BN layer.

Defects can be present at different lattice sites. Here, we would like to examine the effect of defect location on the failure start time and location. To do so, we choose the sample with 10 h-BN layers, and insert only one B vacancy into only one of the 10 h-BN layers. The variation of failure start time t_F_ with the defect position L_V_, where L_V_ is the number of layer counting from the top layer, is shown in Fig. 5. It is seen that the defect location has a great effect on the failure start time. For example, when the defect is located at the top layer, the failure start time is 87.5 ps, which is much lower than the defect-free sample,135.75 ps. With the increase of L_V_, the failure start time increases. For example, at L_V_ = 3, the failure start time increases to 99.3 ps, while at L_V_ = 9, the failure start time increases to 115.25 ps, which is only slightly lower than the defect-free counterpart. The above results suggest that the closer the defect to the top layer is, the earlier the failure start time is. The snapshots of the failure processes for L_V_ = 1, L_V_ = 3, and L_V_ = 9 are shown in Fig. 6a1–a4, b1–b4, c1–c4, respectively. It is seen that the failure starts all from the defect, suggesting that the defect is the weakest link. However, for L_V_ = 1, it is seen that once the failure starts (Fig. 6a2), the failure propagates outwards along the first layer (Fig. 6a3). Figure 6a3,a4 show that vaporization occurs during the propagation and the failure occurs only within the first layer (Fig. 6a4) with the time up to 89.25 ps. For L_V_ = 3, once the failure starts from the defect site (Fig. 6b2), the failure also propagates outwards, and is confined within the layer. At about 100 ps, the 4th layer also starts to fail (Fig. 6b3). During the propagation, melting and vaporization occur, causing the swelling of the top two layers. At 100.75 ps, the top 2 layers and the 5th layer also start to fail. As a result, a crater is formed. For L_V_ = 9, once the failure starts from the defect site (Fig. 6c2), the failure also propagates outwards and is confined within the layer (Fig. 6c3). During the propagation, melting and vaporization also occur, causing the h-BN film swelling. At about 116.75 ps, the neighbouring layers also start to fail, and the swelling of the film is more evident (Fig. 6c4). Clearly, the position of the defect not only affects the failure start time, but also the failure modes.

The underlying mechanism for the defect location-dependent breakdown can be explained by the competition between energy accumulation and dissipation. At the defect site, due to phonon localizations and scatterings, heat can be quickly accumulated. Depending on the location of the defect, the heat dissipation can be different. On one hand, when the defect location is near the surface, the heat dissipation at the defect site should be slow, leading to an earlier breakdown. One the other hand, when the defect is near the substrate (graphene), due to the high interfacial thermal conductance between graphene and h-BN and ultra-high thermal conductivity of graphene, the heat dissipation at the defect should be fast, leading to a delayed breakdown.

### Defect distribution

**Figure 7:**
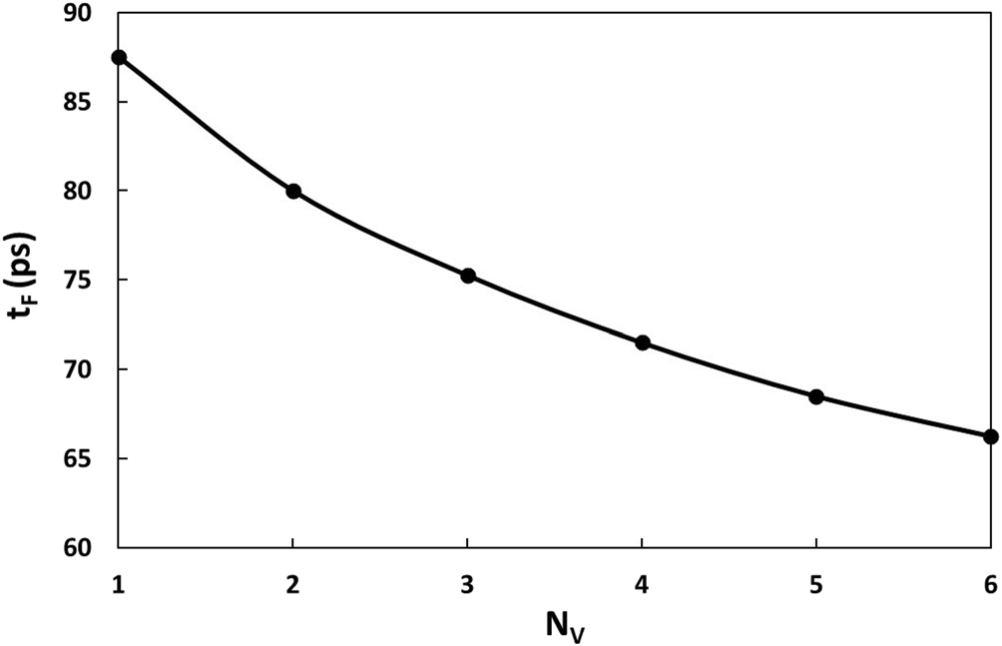
Variation of failure start time with the B vacancy distributions. N_v_ is the number of layers with each containing a B vacancy. The counting of the layers starts from the top layer.

**Figure 8:**
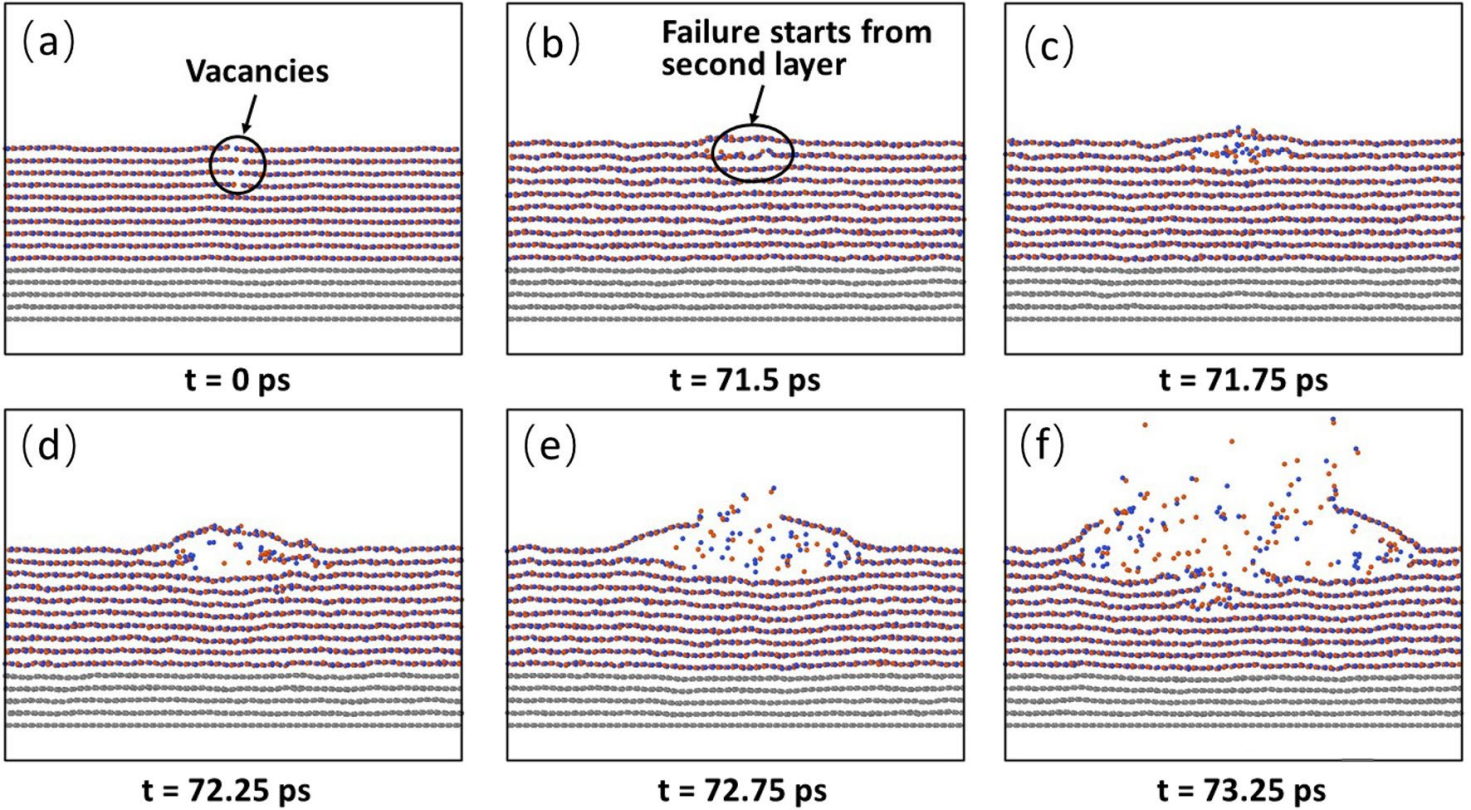
Failure process of the sample with 10 h-BN layers with first to fourth layer each containing a B vacancy. The failure starts from the defect site at the second layer and propagate horizontally and vertically, causing the formation of a pit.

Next, we would like to examine the presence of multiple defects in the h-BN films. Here, we first choose N_V_ layers from the top and insert only one B vacancy into each of the N_V_ layers. The variation of failure start time with N_V_ is shown in Fig. 7. It is seen that the more layers contain defects, the shorter the failure start time is. For example, when only the top layer contains a B vacancy, the failure start time is 87.5 ps. When each of the first two top layers contains a B vacancy, the failure start time decreases to 80 ps. When each of the 4 top layers contains a B vacancy, the failure start time decreases further to 71.5 ps. When each of the 6 top layers contains a B vacancy, the failure start time decreases further to 66.25 ps. Our simulations indicate that the failure can start from any of the defects, and thus shows certain randomness. However, the reduction in the failure start time indicates that the defects at different layers can interact with each other, leading to the reduced failure start time. The interaction can be seen from the snapshots of the failure process of N_V_ = 4 (Fig. 8), where each of the top 4 layers contains a B vacancy. At 71.5 ps, the failure starts from the defect site at the second layer (Fig. 8b). Subsequently, the failure propagates along the second layer. At 72.75 ps, the first layer fails at the defect site (Fig. 8e). At 73.25 ps, the failures at 3rd, 4th and 5th layers also occur (Fig. 8f).

The underlying mechanism for the effect of defect distribution lies in the defect interactions. When two defects are far away from each other, no interactions are present, and each can be treated as an individual defect. However, when two defects are close to each other, interactions occur. Such interactions can intensify the heat accumulation, leading to an earlier breakdown. Hence, when defects are close to each other to form clusters, their interactions can reduce the failure start time, thus accelerating the overall film failure process.

### Boundary effect

**Figure 9:**
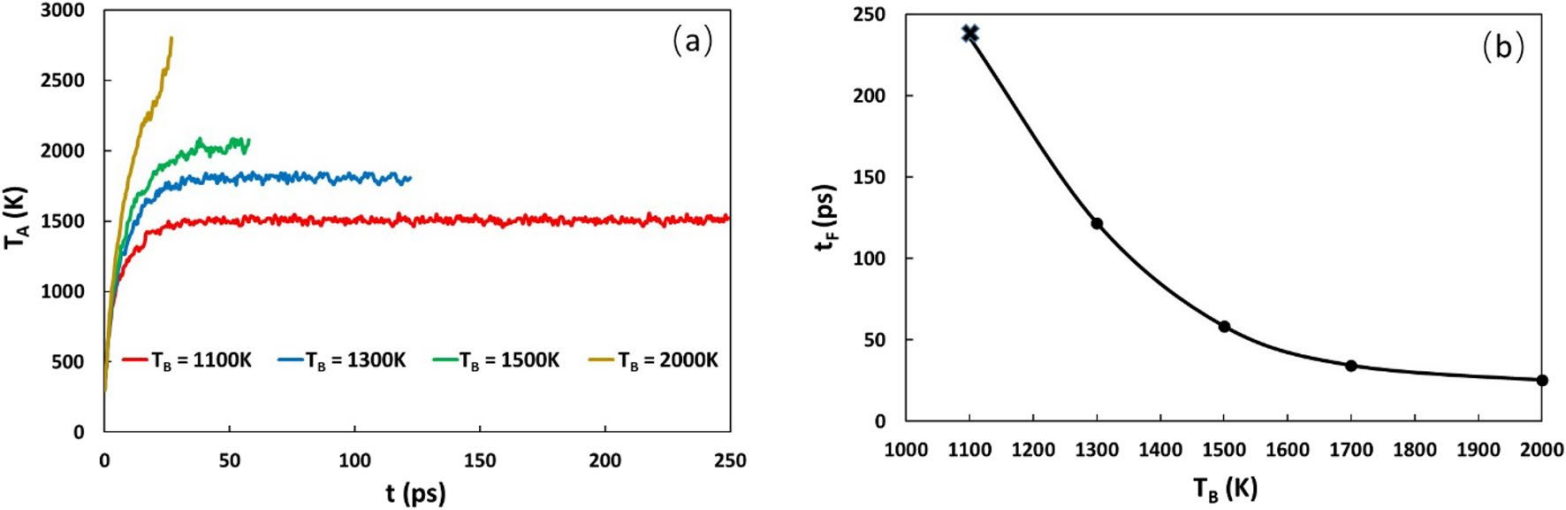
(**a**) The average temperature in the heating zone with the heating time at different fixed boundary temperatures. (**b**). The failure start times at different fixed boundary temperatures. Here the sample with 10 h-BN layers is used. One B vacancy is introduced in the middle of the 3rd h-BN layer. The heating rate is 0.254 eV/atom/ps.

In all above simulations, periodical conditions along the lateral directions are used. During the heating, temperature at the periodical boundaries will increase, and thus the temperature diffusion driving force is decreased, which may cause a shorter failure start time. To examine the boundary effect, we prescribe the lateral boundaries with a fixed temperature, T_B_, to simulate the heat draining at the boundaries. In the simulation, the sample with 10 h-BN layer is used. The heating rate is taken as 0.254 eV/atom/ps, and a B vacancy is inserted in the middle of the 3rd layer. The changes of the average temperature T_A_ within the heating zone with time at the fixed boundary temperature of 1100 K, 1300 K, 1500 K and 2000 K are shown in Fig. 9a and the variation of the failure start time with T_B_ is shown in Fig. 9b. It is seen that at low T_B_, for example, T_B_ = 1100 K, the average temperature reaches a plateau of about 1500 K, no failure occurs up to 250 ps. For T_B_ = 1300 K, although the average temperature appears to reach a plateau, failure still occurs at the defect site at 125 ps. Further examination shows that local heat fluctuations at the defect site cause the local temperature rise, leading to the failure. At T_B_ = 1500 K, no plateau is observed for the average temperature in the heating zone, and failure occurs at the defect site at about 58 ps. For T_B_ = 2000 K, the temperature rises rapidly, and failure occurs at the defect site with a short failure time of about 26 ps.

The underlying mechanism for the boundary effect lies in the competition between heat accumulation and heat dissipation in the heating zone. When the fixed boundary temperature is low, a large temperature gradient can be built up, which can lead to a large heat flux to dissipate the heat out of the heating zone. When the heat flux can balance the heat injection rate, the maximum temperature in the heating zone can no longer increase, leading to a steady state. If the maximum temperature is lower than the melting temperature of h-BN, then breakdown will not occur. The above study suggests that efficient heat draining is important to avoid failure of h-BN during heating.

## Discussion

In the introduction, we have discussed the failure processes and mechanisms of h-BN under an electron beam from several previous studies. These studies clearly show the influences of film thickness and vacancy in h-BN on the structural failure and dielectric breakdown. Of particular interest is the revelation of conflicting results on the failure processes and mechanisms in h-BN under electron beams. Below, we would like to discuss our simulation results in the context of these existing works, aiming to shed some light on these conflicting results.

### Role of defect

Our simulations clearly demonstrate the importance of defect, which can significantly reduce the failure start time and change the failure mode, which may explain different observations in previous experimental studies^10,13,14^.

It is known that the defect generation is generally more difficult than the defect migration and propagation^35^. For example, the defect formation energy for B vacancy is 7.65 eV^33^ while the migration energy for B is only 2.6 eV^34^. In addition, in forming a B vacancy, rapture of three B–N bonds is required, while for failure propagation from a B vacancy, rapture of only two B–N bonds is required. This difference explains the large difference in the failure start time with and without B vacancy.

Previous experimental studies have shown that defects play an important role in the dielectric breakdown^11,13,14^. In particular, it was found that B vacancies were the critical defect type responsible for the breakdown^11^. It was also found that the “defect generation” was not random but occurred preferentially at “specific locations”^13^. Our simulations show that when a B vacancy is present in the heating zone, failure always starts from the defect and propagates from there. Hence, these “specific locations” revealed in Ref.^13^ are likely the existing B vacancies. Since it is difficult to nucleate a vacancy due to the higher energy cost^33^, and vacancy defects cannot be avoided^15,16^, it is likely that it is those existing defects rather than the newly created defects that are responsible for the initiation of the breakdown failure.

**Figure 10:**
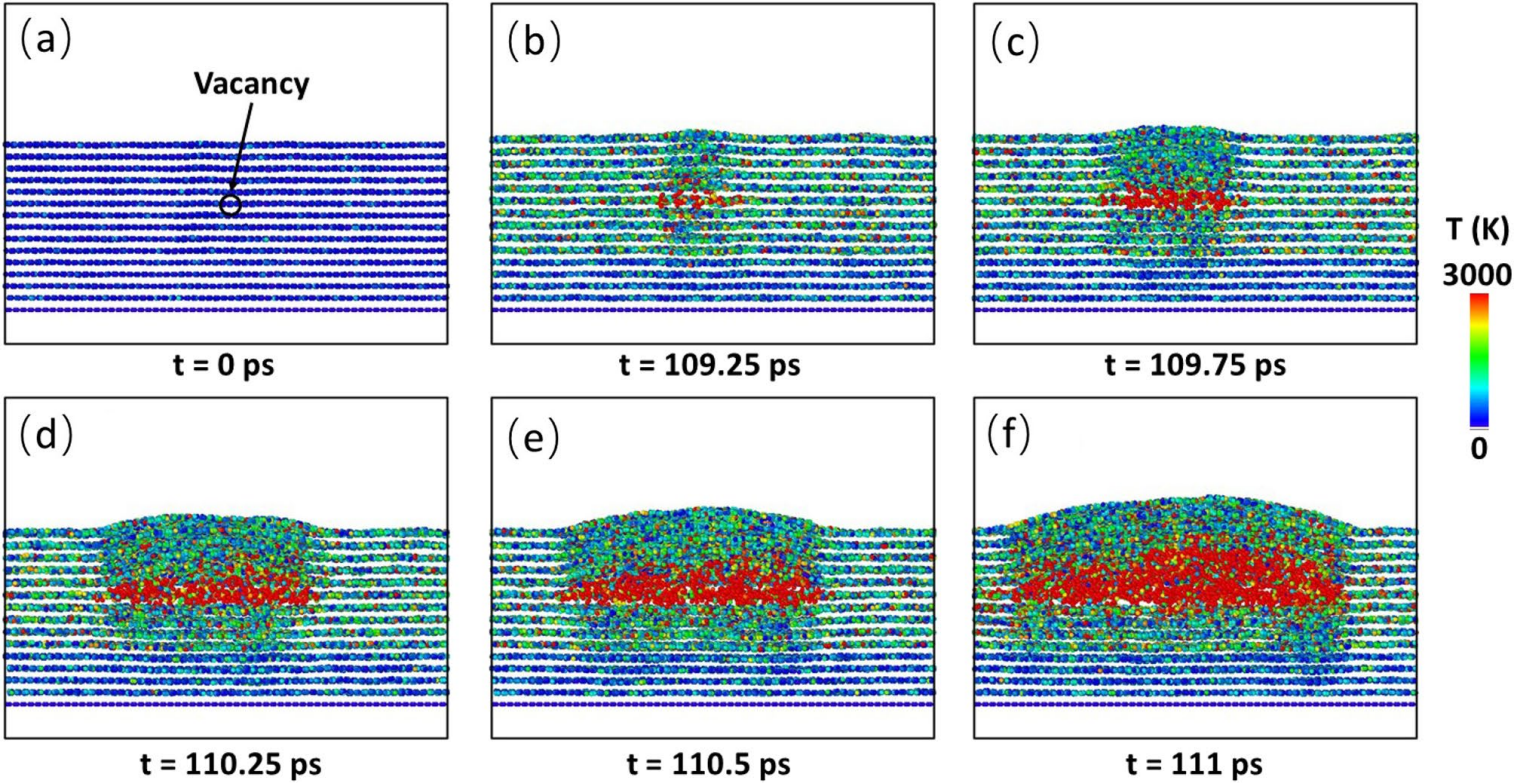
Snapshots of atomic temperature distributions in the 10-layer h-BN sample. The heating rate is 0.254 eV/atom/ps. One B vacancy is introduced into the middle of the 6th layer.

Our simulations show that the temperature at the defect can be significantly higher than its surrounding, which can be clearly seen from Fig. 10 for the 10 h-BN layers with a B vacancy at the middle of the 6th layer. The heating rate of 0.254 eV/atom/ps is used and periodical boundary conditions is applied. It is seen that there is a heat accumulation at the defect site (Fig. 10b), causing a significant temperature rise at about 3000 K. This is consistent with the understanding that a defect can cause phonon localizations. When the failure propagates, local melting and vaporization occur along the 6th layer, causing a significant temperature rise in the failure zone, while the temperature at its surrounding is much lower. This can be explained by the melting and vaporization processes. It is known that both melting and vaporization require energy inputs since the enthalpy of liquid and vapor are different from that of solid phase. The heat generated by the heating can be efficiently absorbed by the phase transformation from solid to liquid and vapor.

For multiple defects, our study shows that each defect can cause phonon localizations and heat accumulation at the defect site. When the phonon localization zones of two defects start to overlap, this can cause a temperature rise at the defects. Clearly, the interactions and couplings of these nearby defects lead to a shorter failure start time in samples with multiple defects. Previous studies^10,11^ have suggested that defect clusters arising from microstructural inhomogeneities, such as grain boundaries and vacancy clusters, can be a prominent reason for the dielectric breakdown. This is consistent with our study here, which suggests that vacancy interactions can significantly reduce the dielectric strength of h-BN.

### Effect of layer number and beam power

In the introduction, we have shown that some studies observed a layer-by-layer breakdown^9,10^, others observed hole or pit formation^9^, while some others showed surface extrusions or hillocks of h-BN films^12,13^. Also, failure patterns in monolayer (without hillock) and multilayer (with hillock) were found to be different^12^. Below, we would like to discuss the possible underlying reasons for these differences.

Previous study^12^ has shown that under c-AFM, extrusions or hillocks appeared on the surface of all the h-BN samples when failure starts, except in monolayer h-BN. In our simulations, all the multiple layer h-BN samples (with or without defect) show swelling at the beam spot and hillock formation at the surface. For monolayer h-BN, once failure starts, it will propagate along the layer through vaporization, without the formation of hillock. Hence, our simulations are consistent with the above experimental results^12^.

We also note that pits or holes were observed in experiments using c-AFM^9^. Our simulations show that in multilayer h-BN samples, when defects are located at the heating zone, a pit can be formed. However, before the formation of a pit, a hillock should be formed first. This is because the large pressure caused by melting and vaporization of h-BN layers at the defect site underneath the surface causes the layers above to burst, leading to the formation of a pit. Clearly, for multilayer h-BN samples, if the failure starts underneath the top layer, a hillock occurs first. With continued heating, the formation of a pit will then follow. Our simulation may explain why in some cases, hillocks are observed while in other cases, pits or holes are observed^9^.

In our above simulations, we have assumed that heating occurs within a cylindric zone with a radius of 1 nm and the depth equal to the h-BN film thickness. In reality, the lateral size and depth of the heating zone may depend on the beam power and beam focus. When the beam focus area is fixed, with the increase in the heating power, it is expected that the higher energy phonons will be generated. Since the phonon energy is ħω, where ħ is the reduced Planck constant and ω is the phonon frequency. This indicates that the higher the phonon frequency, the higher the phonon energy. The heat diffusion length can be written as^36^: ${L}_{D}=\sqrt{2\alpha /\omega }$, where α is the thermal diffusivity and the heat travel speed is $V=\sqrt{2\alpha \omega }$. It can be deduced that higher frequency or energy phonons propagate faster but nearer while lower frequency or energy phonons propagate slowly but farther. When the beam power or frequency is high, the phonons can only travel in a short distance, and the energy will dissipate primarily with the top layers. As a result, a layer-by-layer failure could occur in the h-BN film^9,10^. Interestingly, the layer-by-layer failure could also result in the formation of a pit^9^. On the other hand, when the beam power or frequency is not high, the beam energy can penetrate deep but travel slowly into the h-BN film. In this case, failure could start inside the film, resulting in the defect formation inside the film and subsequent swelling of the film. The above analysis is consistent with the experimental observation in Ref.^13^.

## Conclusion

In this work, we perform molecular dynamics simulations to examine the failure processes and mechanisms of h-BN films by varying their thickness and defect concentration and distribution under local energy injection. Due to the local energy injection, the atoms within the heat injection zone absorb the energy, and the absorbed energy is then turned into heat. Our simulations show that for defect-free h-BN films, the failure start time decreases with the increase in the film thickness under the same energy injection rate. In the presence of atomic defects, the films always start the failure at the defect sites in the energy injection zone. When the defect sites are away from the surface layer, surface extrusion occurs due to the melting or vaporization of the internal layers. With continued heating, rupture of the top layers could occur, which could cause the burst of the h-BN layers above, causing the formation of pits. However, when the defect density is high and defects distribute in different layers, defect interactions and couplings can occur, which further reduces the failure start time. For the monolayer h-BN, the monolayer could fail at either pre-existing defect site or through the creation of defects, and melting or vaporization could occur, without going through the swelling. We also compare our simulation results with existing experimental observations, and remarkably, our simulations reproduce and explain several different failure modes observed in previous experiments. The present work not only reproduce several interesting experimental observations, but also reveal interesting mechanisms underneath these observations. It is expected that these findings and understandings will provide new strategies to control the h-BN failure processes and avoid heating breakdown of h-BN films.

## Data Availability

The datasets generated during and/or analysed during the current study are available from the corresponding authors on reasonable request.
